# Supplementary material for: Are the forearm muscles excited equally in different, professional piano players?
Source: PLoS One. 2022 Mar 22;17(3):e0265575. doi: 10.1371/journal.pone.0265575 (PMC8939780; doi:10.1371/journal.pone.0265575)
Supplement: S1 Appendix — (DOCX) [file pone.0265575.s001.docx]

**Title**: Forearm muscle excitation analysis for different professional piano players.

**Authors**: Alba Thio-Pera^1¶*^, Matteo De Carlo^2,3^, Andrea Manzoni^4^, Fabrizio D’Elia^4^, Giacinto Luigi Cerone^1,2^, Giovanni Putame^2,3^, Mara Terzini^2,3^, Marco Gazzoni^1,2^, Cristina Bignardi^2,3^ and Taian Vieira^1,2¶^

^1^ Laboratory for Engineering of the Neuromuscular System (LISiN) – Politecnico di Torino, Turin, Italy

^2^ PolitoBIOMed Lab, Politecnico di Torino, Turin, Italy

^3^ Department of Mechanical and Aerospace Engineering, Politecnico di Torino, Turin, Italy

^4^ Another Music Records, Paris, France

*** corresponding author**: [thioalbs@gmail.com](mailto:thioalbs@gmail.com) (AT)

# Appendices

## **Experimental instructions for Octaves tasks.**

The experimental instructions were provided in Italian, since all the professional pianists that participated on the study were Italians. However, an English translation has been added at the end of every paragraph.

**Task 1: Ottave di avambraccio.**

Istruzioni: L’azione dell’avambraccio è il motore di questo movimento. Il movimento dell’avambraccio è provocato dall’alternanza del muscolo tricipite e bicipite che durante un’azione pianistica vanno sollecitati in alternanza.

*Instructions: The action of the forearm is the engine of this movement. The movement of the forearm is caused by the alternation of the triceps and biceps muscles which must be solicited alternately during a piano action.*

**Task 2: Ottave di polso.**

Istruzioni: Il movimento del polso è causato dall’azione dei flessori e degli estensori, cioè quei muscoli che hanno l’azione di sollevare ed abbassare il palmo della mano. Questo movimento può essere attivo o passivo.

*Instructions: The movement of the wrist is caused by the action of the flexors and extensors, that is, those muscles that have the action of raising and lowering the palm of the hand. This movement can be active or passive.*

**Task 3: Ottave di dito.**

Istruzioni: per le ottave di dita il movimento fondamentale è l’estensione e la flessione di esse nel rispetto della leva del dito e del fondo del tasto del pianoforte (cioè il punto d’appoggio nel quale il dito scarica il suo peso).

*Instructions: for the octaves of fingers, the fundamental movement is the extension and flexion of them in respect of the finger lever and the bottom of the piano key (i.e. the point of support where the finger unloads its weight).*

**Task 4: Ottave spezzate.**

Istruzioni: utilizzo del movimento di rotazione (pronazione e supinazione) dell’avambraccio.

*Instructions: use of the rotation movement (pronation and supination) of the forearm.*

***Experimental instructions for classical tasks.***

**Task 5: Schicksal in Arbeit (Arr. Andrea Manzoni)**


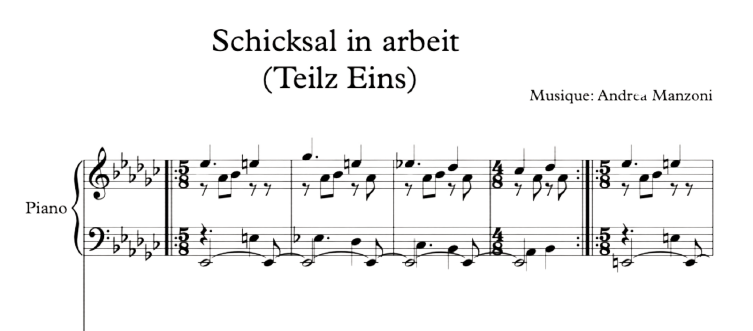


Istruzioni: suonare le prime 4 battute in loop per 4 volte. In esecuzione stare vicinissimi ai tasti utilizzando il maggior risparmio energetico possibile.

*Instructions: play the first 4 measures in a loop 4 times. When playing, stay very close to the keys using the greatest possible energy saving.*

**Task 6: Rachmaninov - Preludio in C#min op.**


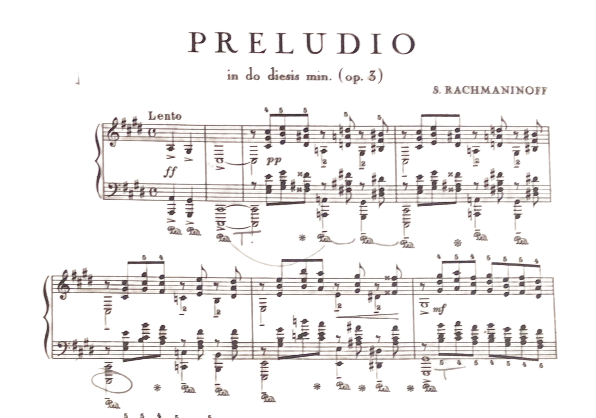


Istruzioni: Movimenti di reazione del polso e braccio. Suonare le prime due righe del brano. In esecuzione stare vicinissimi ai tasti utilizzando il maggior risparmio energetico possibile.

*Instructions: Reaction movements of the wrist and arm. Play the first two lines of the song. When running, stay very close to the keys using the greatest possible energy saving.*

**Task 7: Schubert - Improvviso Op.9 n. 2**


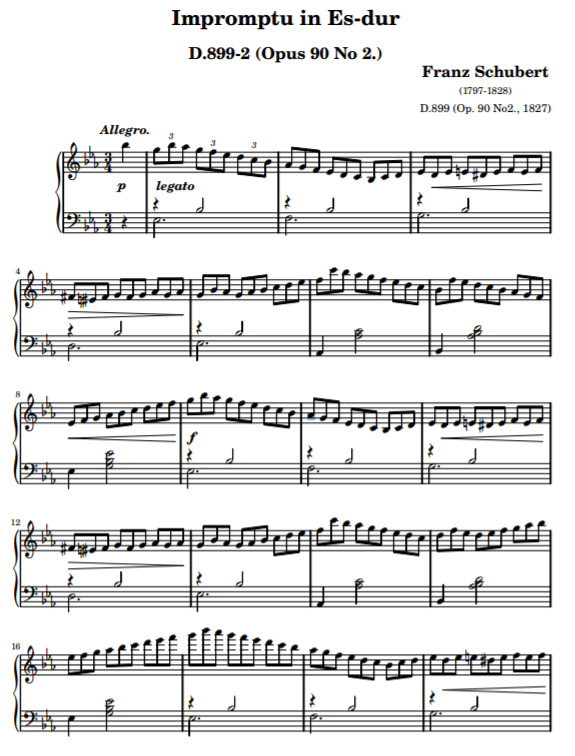


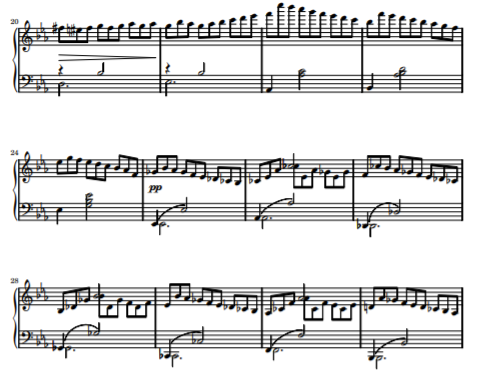
Istruzioni:

- eseguire la prima pagina

- suonare alla velocità che si desidera e che sia il più confortevole possibile

- scegliere la diteggiatura che si preferisce

- suonare il brano scegliendo la dinamica e la tipologia di esecuzione a piacimento

*Instructions:*

*- play the first page*

*- play at the speed you want and that is as comfortable as possible*

*- choose the fingering you prefer*

*- play the piece by choosing the dynamics and the type of execution at will*

**Task 8: Improvisation for 5 min.**

Istruzioni: l’improvvisazione sarà completamente libera. Potrete eseguire gli stili che vorrete senza alcun vincolo. L’importante è che all’interno della performance siano presenti passaggi a note singole e passaggi accordali.

*Instructions: the improvisation will be completely free. You can perform the styles you want without any constraints. The important thing is that within the performance there are single note passages and chord passages.*
