# Supplementary material for: Are the forearm muscles excited equally in different, professional piano players?
Source: PLoS One. 2022 Mar 22;17(3):e0265575. doi: 10.1371/journal.pone.0265575 (PMC8939780; doi:10.1371/journal.pone.0265575)
Supplement: Attachment — Submitted filename: rebuttal-letter.docx [file pone.0265575.s002.docx]

Reviewer #1

Thank you for the opportunity to review this manuscript, “Are the forearm muscles excited equally in different, professional piano players?”. This study tests the hypothesis that regions of strong forearm muscle activity during dynamic piano playing will be similar across a cohort of experienced pianists. Results are consistent with the hypothesis, showing pronounced dorsal (i.e., extensor) muscle activity when playing octaves and pronounced ventral (i.e., flexor) muscle activity when playing classical/jazz pieces and improvising.

I really enjoyed reviewing this, both as a biomechanist and an amateur pianist myself. I think the approach is interesting and that the methods are sound. Given the broad appeal of music outside of scientific circles alone, I expect this study could generate broad interest. However, I have a few concerns that should be addressed prior to publication.

**Thank you very much for taking the time to review our paper and for your comments. We are glad that you enjoyed the reviewing and that you found it interesting. We have addressed each of your comments and suggestions in turn. To facilitate reading, we used red text to highlight all changes made to the manuscript.**

The primary finding of this study is that professional pianists show strong muscle activity in the region of the forearm roughly corresponding to the medial and lateral epicondyles – in other words, roughly corresponding to the common flexor and extensor mass, respectively. My concern is that given the limited proximo-distal spread of the electrodes, I’d be surprised if the authors found anything else – i.e., for the proximal forearm region sampled, the most likely outcome would be high activity in the common flexor and extensor masses. I know there are technical difficulties in adding more electrodes, or – alternatively – tradeoffs between sampling breadth and resolution if the electrode cuff were spread across the entire forearm. Nonetheless, I think some discussion about the possible limitations of ONLY sampling in the proximal forearm would be warranted.

**Thank you for these observations. We amended discussion accordingly (Lines 465-473).**

Additionally, the manuscript begins with a discussion of playing related musculoskeletal disorders (PRMDs), partly as a justification for undertaking the study. Is it possible, in the discussion, to relate the findings of this study with particular PRMDs? For instance, do pianists get tendinitis in the regions identified as major centres of muscle activity?

**Thank you for this comment. We added two sentences in Discussion (Lines 442-446) in response to this comment. We haven’t found any study or publication that goes into that much depth for the specific PRMDs forearm regions. It has been stated in the literature that the forearm (in general) is one of the most important locations causing PRMDs. However, there is a dearth of evidence on the specific forearm regions where PRMDs may take place. Even though we mention in Discussion that the forearm sites frequently excited could also be the sites where PRMDs are more likely to manifest, this remains to be proven. We expect that the results of this study can help furthering our understanding on the incidence of PRMDs.**

Data availability - The authors state that project data are within the manuscript and supporting information files. However, I did not see raw project data in either place. As stated in the PLOS Data Policy, “Authors are required to make all data underlying the findings described fully available, without restriction, and from the time of publication.”.

**Thank you for this observation. The data has now been updated to Figshare and it is now available from the Figshare database (DOI: 10.6084/m9.figshare.18753014)**

(Lines 94-103) Was there any effort to gauge the musculoskeletal health of the participants (i.e., did they have a histology of PRMDs, or any current pain or other issues)?

**We did collect this information, through a self-reporting questionnaire though. Questions 1, 4, 6 and 7 in the subject form we attach for the reviewer information specifically deal with PRMDs and pain. We slightly modified lines 99-100 to clarify this point.**

(Line 117) I believe the correct English translation for spezzate would be “broken octaves”. Would this be an example of what the pianists were required to do? <https://youtu.be/cUOQJ7ukYrk?t=545>

**No, the example is not correct. The pianist uses the "forearm octave" in the video. An example of the broken octave is illustrated in the following vide - from 1:27 to 1:39**

[**https://www.youtube.com/watch?v=MF7U1QYS1zE&ab_channel=Musicnotes**](https://www.youtube.com/watch?v=MF7U1QYS1zE&ab_channel=Musicnotes)

(Lines 122-124) Were pianists given any instructions on tempo for the classical/jazz pieces, or just told to play as slow/fast as they wished? Also, the standard English translations for the classical pieces would be “Prelude in C-sharp minor of Rachmaninov” and “Impromptu Op. 90 No. 2 of Schubert” – Opus 9 is a collection of dances. Finally, isn’t Andrea Manzoni the composer of Schicksal in Arbeit?

**Pianists played at their preferred tempo; it has been added on the text. Thank you, the translations of the pieces have been updated. Indeed, the composer of Schicksal in Arbait is an original composition for piano of Andrea Mazoni, this has also been updated (lines 121-123).**

(Lines 149-150) Please indicate here that the forearm was in pronated position when the length measurement was taken. I was trying to figure out why length was defined as lateral epicondyle to ulnar styloid process (i.e., diagonally across the forearm) until I looked at Figure 1 and saw the forearm was pronated.

**Yes, thank you, we have added it to the text (line 150).**

(Discussion) In thinking about the different EMG results for octaves versus more melodic pieces, I wondered if the results in both cases said more about isometric/eccentric muscle contractions, rather than concentric muscle contractions per se. For instance, playing octave runs requires the pianist to use lots of shoulder and elbow joint movement while keeping the wrists relatively rigid. In this case, one might expect strong activity across the entirety of the forearm muscles to maintain a stable (isometric) position of the wrist. Conversely, playing melodic pieces requires lots of independent finger movement and a more supple wrist (though this varies of course, depending on the piece and the style). Nonetheless, I would expect strong eccentric flexor activity to make sure the wrist does not collapse into extension from the reaction force of pressing the keys. Perhaps a discussion along these lines could be added to the Discussion section of the manuscript.

**Thank you for this contribution. A new paragraph has been added on the discussion to include these points (Lines 403-414).**

(Supplemental Information) Please provide and English language translation of the instructions. I used Google translate, and it seemed to work well.

**We first decided to provide the instructions in Italian because it was how information was presented to all the musicians. We agree however with the reviewer that it would be beneficial for readers to have instructions translated. We have added an English translation as suggested.**

Reviewer #2

In this study, Thio-Pera and colleagues studied excitation in a grid of EMG electrodes of eight professional piano players playing a mix of carefully chosen pieces. I think this is a fascinating piece that may be of interest to diverse audiences (musicians, healthcare providers and anatomists), and therefore, PLoS ONE is a great journal choice. Although I believe that this piece is essentially publishable as is, I have one caveat and two suggestions:

**Thank you very much for taking the time to review our paper and for your comments. We are very glad you find it interesting and fascinating. We have addressed your two suggestions by separating the main questions. To facilitate reading, we used red text to highlight all changes made to the manuscript.**

Caveat: the statistics went WAY above my head. I really do not understand these analyses and have no clue what “pseudo t values” are. Although the results and discussion thereof seem plausible to me, if someone with more familiarity with these analytical methods calls them into question, I defer to that opinion.

**We tried to explain the statistical process as clearly as we could. The process we followed is the third example detailed at Nichols et al. 2001 and, for this reason, we did not want to focus our article on that so to not confuse the reader. In agreement with the reviewer comment we attempted to clarify the concept of pseudo t values at lines 251-252. The pseudo t value is mainly a t value only that instead of using the standard deviation values we use a smoothed standard deviation. Smoothing is necessary to contend with near-zero variances that may arise just by chance, inflating the t-statistic images and therefore Type I error. In our case, physiological considerations suggest that the variance can be constant in small localities and so smoothing the pixel variances with its neighbors can give a better estimation of the actual variance.**

Suggestion 1: I think that the figures in general could use some refinement. Specifically, Figure 1 needs more explanation and does not seem to fully agree with what is written about these methods in the text. I would request that the authors review the methodological description of electrode placement and make sure that it is clearly in accordance with this figure (e.g., I don’t really understand where any but the reference electrodes were placed or where the “circumference of the forearm was measured at 10% of its length”). If it is discussed in the methods, could the authors please show it in the figures?

**Thank you for noticing this, the forearm circumference was measured at the 20% of the length (this has been updated on the text), where the first line of electrodes was to be placed. This is marked on the figured with a dotted line with the 0.2 l. The reference electrode was placed at the medial epicondyle as written on the line 162-163 but it was indeed not noted on the image. The image has now been updated accordingly.**

The other figures are even more confusing. Are these heat maps of one individual? Playing one piece? A combination of all of the individuals?

**For figure 2 the heat map corresponds to Subject 4 playing the third octave, in accordance with the title of the figure. We acknowledge that it is not stated elsewhere the numeration of the octaves, so we have updated the figure changing octave 03 with finger octave at slow speed.**

**For figure 3 the example is shown for improvisation task. We didn’t specify the task since we were trying to illustrate the statistical process itself and the same has been followed for all the different tasks. In agreement with the reviewer suggestion, we have added on the image caption that the example is shown for improvisation task. The first step shows the example for the first subject as mentioned in the subtitle, whereas all subjects are included in the other steps. We understand Figure 3 is dense, but we believe it may help readers in understanding the statistical analysis applied for the first time to EMG images.**

**Figure 4 shows the result of the permutation test across subjects and the titles of each heat map state which piece is played. Not sure if we can add anything else to clarify this point.**

**Figure 5 shows the result across subjects and tasks. Again, not sure if we can add anything to clarify this point.**

**We are committed with making figures that greatly help readers in grasping the concept we wish to transmit. Should the reviewer feel any specific modification would be necessary for our goal to be reached, other than those we implemented following the reviewer comments below, please kindly inform us.**

Figure 3 is especially confusing. It seems that this is a whole explanation of a method, but this is not spelled out really in the text or in enough detail in this figure.

**In agreement with the reviewer comment, we modified the caption of Figure 3 to explicitly indicate it illustrates the procedures requested for applying the permutation statistical test to EMG images (lines 227-231). We did that also in the text (lines 236-237), where we explain the method step by step.**

Although figure 5 is a bit clearer than most, it is a prime example of how the figure does not match the text: “centering single pairs of electrodes at roughly 15% and 75% of the forearm circumference, 35% distally to the elbow joint”. None of these values are actually shown as landmarks on the figure. This would be an easy addition that would very much help readers understand.

**Thank you. We modified Figure 5 accordingly. Based on our results we feel it would be not accurate to provide a pointed indication where to centre a pair of bipolar electrodes. For this reason, we used large circles to indicate the region we recommend for centring a pair of electrodes. We also modified the text in discussion (Lines 457-459) to specifically mention our recommendation takes into account the different sizes of the commercially available electrodes used for surface EMG recording.**

Suggestion 2: As an anatomist, I would really appreciate deeper discussion of the EMG results from an anatomical perspective – maybe even only one more discussion paragraph? Aside from broad statements about flexor vs. extensor compartments, could the authors discuss differences in medial or lateral activation?

**Thank you for the kind suggestion. We have added a new discussion paragraph to associate the results with piano playing movements. In this new paragraph we present a competing explanation for the activation of flexor, forearm muscles during classical playing, in contrast with the notion of efficient, playing style (Lines 403-414).**

**Nonetheless, during this study, we did not attempt to target specific muscles on the forearm. Due to the difficulty of isolating individual forearm muscles, we used high-density EMG to investigate regional excitation with respect to anatomical landmarks. This has led us to identify some regions which are more likely to represent the forearm muscle activity while playing piano. We find it hard to associate these regions with specific muscles. However, we can generally associate the ventral region of the grid with the flexor muscle masses and accordingly the dorsal with the extensor muscle masses.**

What is the significance of only looking at the proximal portion of the muscles? Might the results have been different at more distal placement of the grid? Speaking of the grid, could the authors add more to the caveat about the use of the static grid? These electrodes would have been measuring very different muscles in the player with the smallest forearm circumference vs. the one with the largest one. As the authors seemingly know, it would have been better to use a grid pattern that scaled the distance between electrodes based on forearm dimensions rather than a static pattern. This might have been achievable with some kind of elastic mount. Unfortunately, that is not the way the data were collected. A few more sentences about the effects of this error (more than just ‘we mostly missed the ulnar bone region in the big guys’) might help future investigators not repeat it.

**Thank you for these observations. In agreement with comments from the two reviewers, we added a few lines of discussion to deal with the relatively short coverage of forearm in the proximo-distal direction (Lines 465-473). We nevertheless have a different view as per the error of using a grid with fixed inter-electrode distance across subjects. We understand different electrodes may have covered different muscle regions, owing to the inter-individual, anatomical variability. By interpolating EMGs, however, we were able to contend with this issue, representing the distribution of the surface electric potential at the same relative sites across subjects. The price we paid for doing so is that we had to limit the interpolated region to that of the subject with the thickest and longest forearm. Within the grid, therefore, we would feel safe to state we are comparing like with like across subjects.**

**We appreciate the concern with the possibility of elastic grids to deal with anatomical differences between subjects. From a technical perspective this is challenging. We made some attempts in this direction, but still with a fix inter-electrode distance. Elastic electrodes would require the conductive paths connecting electrodes to do not break while changing shape to accommodate stretching. Another issue, more conceptual, would be related to how comparable the distribution of electric potential on the skin would be for different subjects. Even though an elastic arrangement could lead to a relative, similar inter-electrode distance across subjects, the spatial filtering properties would change. The changes would unlikely be linear, meaning that differences in the distribution of electric potential on the skin would not scale linearly with the different inter-electrode distance. With our approach we were able to map electric potentials using the same spatial filtering properties and, then, scale the sampled distribution (sampled with a fixed, spatial sampling frequency) to the different geometries. We feel deepening this discussion in the manuscript would be not appropriate.**

**Cerone GL, Botter A, Vieira T, Gazzoni M. Design and Characterization of a Textile Electrode System for the Detection of High-Density sEMG. IEEE Trans Neural Syst Rehabil Eng. 2021;29:1110-1119. doi: 10.1109/TNSRE.2021.3086860.**
